# Structural and Electrical Characterization of Pure and Al-Doped ZnO Nanorods

**DOI:** 10.3390/ma14237454

**Published:** 2021-12-04

**Authors:** Ivana Panžić, Ivana Capan, Tomislav Brodar, Arijeta Bafti, Vilko Mandić

**Affiliations:** 1 Faculty of Chemical Engineering and Technology, Marulićev trg 20, 10000 Zagreb, Croatia; ipanzic@fkit.hr (I.P.); abafti@fkit.hr (A.B.); 2 Ruđer Bošković Institute, Bijenička 54, 10000 Zagreb, Croatia; ivana.capan@irb.hr (I.C.); tomislav.brodar@irb.hr (T.B.)

**Keywords:** ZnO nanorods, n-type doping, chemical bath synthesis, electrical transport mechanism, KPFM

## Abstract

Pure and Al-doped (3 at.%) ZnO nanorods were prepared by two-step synthesis. In the first step, ZnO thin films were deposited on silicon wafers by spin coating; then, ZnO nanorods (NR) and Al-doped ZnO NR were grown using a chemical bath method. The structural properties of zincite nanorods were determined by X-ray diffraction (XRD) and corroborated well with the morphologic properties obtained by field-emission gun scanning electron microscopy (FEG SEM) with energy-dispersive X-ray spectroscopy (EDS). Morphology results revealed a minute change in the nanorod geometry upon doping, which was also visible by Kelvin probe force microscopy (KPFM). KPFM also showed preliminary electrical properties. Detailed electrical characterization of pure and Al-doped ZnO NR was conducted by temperature-dependent current–voltage (I–V) measurements on Au/(Al)ZnO NR/n-Si junctions. It was shown that Al doping increases the conductivity of ZnO NR by an order of magnitude. The I–V characteristics of pure and Al-doped ZnO NR followed the ohmic regime for lower voltages, whereas, for the higher voltages, significant changes in electric conduction mechanisms were detected and ascribed to Al-doping. In conclusion, for future applications, one should consider the possible influence of the geometry change of (Al)ZnO NRs on their overall electric transport properties.

## 1. Introduction

ZnO is a direct wide-bandgap (3.37 eV) semiconductor with a large exciton binding energy of 60 meV, high electron mobility, high breakdown electric field strength, high radiation tolerance, and high thermal conductivity [1,2], suitable for electronic, optoelectronic, and sensing applications. ZnO has a hexagonal wurtzite P63mc structure, where each anion is surrounded by four cations at the corners of a tetrahedron. The tetrahedral coordination is typical for covalent bonding, but ZnO has a substantial ionic character. ZnO, where zinc atoms are present in excess in comparison to oxygen atoms, resides at the borderline between covalent and ionic semiconductors. It is a nonstoichiometric compound due to the excess of zinc atoms, and even undoped ZnO shows intrinsic n-type conductivity with high electron densities around 10^21^ cm^−3^. Zinc interstitials, Zn_i_, and the oxygen vacancies, V, are the dominant donors in undesired n-doped ZnO films. However, this remains controversial since impurities that are shallow donors, such as hydrogen [3], are unintentionally introduced and could cause the abovementioned behaviour.

The synthesis and characterization of one-dimensional (1D) semiconductor materials have recently attracted more interest due to their physical properties that allow them to play an important role in optoelectronic and electronic devices at the nanoscale. A com-bination of the three types of fast growth directions (〈2110〉, 〈0110〉, and 〈0001〉) and the three area-adjustable facets ({2110}, {0110}, and {0001}) of ZnO leads to the growth of a di-verse group of hierarchical and complicated nanostructures [4]. This is partially reflected in the many different structural configurations of ZnO nanomaterials such as nanowires, nanorods, nanotubes, nanobelts, nanorings, and nanosprings, which are attractive for a variety of applications [5]. In particular, ZnO nanorods (NRs) are being investigated for solar cells, photoluminescence devices, and sensors [6]. The large surface-to-volume ratio provides a large active area, which is advantageous for gas sensors, while the high electron mobility and transparency are valuable for electron transport layer in solar cells [7,8]. Growth of ZnO nanorods can be achieved using techniques such as metal organic chemical vapor deposition [9], thermal evaporation [10], radiofrequency magnetron sputtering [11], pulsed laser deposition [12], and spray pyrolysis [13] (Table 1). These techniques have been used successfully; however, many of these require specific conditions for successful reaction, such as high temperature, high pressure, and an inert atmosphere. On the other hand, chemical solution processes, e.g., chemical bath deposition, greatly facilitate the fabrication of well-aligned ZnO NRs on a large scale at low temperatures.

Precoating the substrate with a ZnO seed layer is crucial for the subsequent growth of highly oriented ZnO NR arrays from the solution [14]. Control over the nanorod growth can be gained simply by varying the layer thickness, layer patterns, and deposition techniques [15]. Hexamine, also known as hexamethylenetetramine (HMT), is a highly water-soluble, nonionic tetradentate cyclic tertiary amine. Thermal degradation of HMT releases hydroxyl ions which react with Zn^2+^ ions to form ZnO [15]. The aqueous solutions of zinc nitrate and HMT can produce the following chemical reactions:

(CH_2_)_6_N_4_ + 6H_2_O ↔ 6HCHO + 4NH_3_, (1)

NH_3_ + H_2_O ↔ NH_4_^+^ + OH^−^, (2)

Zn(NO_3_)_2_ + 6H_2_O → Zn^2+^ + 2(NO_3_)^−^ + 6H_2_O, (3)

Zn^2+^ + 2OH^−^ ↔ ZnO + H_2_O. (4)

The consensus is that the role of HMT is to supply the hydroxyl ions which drive the precipitation reaction [16]. Initially, due to the decomposition of zinc nitrate hexahydrate and HMT at an elevated temperature, OH^-^ is introduced into the Zn^2+^ aqueous solution.

In the hydrothermal growth process, with the temperature increase, the concentrations of Zn^2+^ and OH^−^ also increase. When the concentration increases to a critical value of solution supersaturation, spontaneous fine ZnO nuclei are formed. Furthermore, the formed nanoparticles combine, reducing the free interface energy [17]. Due to the higher symmetry of the (001) plane compared to other planes along the c-axis, it is the typical growth plane. Nucleation determines the surface-to-volume ratio of the ZnO NR. Subsequently, the incorporation of growth units into the crystal lattice of the nanorods by dehydration takes place.

The electrical, optical, and mechanical properties of ZnO NR can be improved by doping [18,19,20,21,22,23,24]. N-type doping is relatively easy in comparison to p-type doping. Group III elements such as Al, Ga, and In as substitutional elements for Zn and group VII elements such as Cl and I as substitutional elements for O can be used as n-type dopants. Among possible dopants, Al is particularly interesting due to the enhancement of conductivity and optical bandgap [25]. Al doping introduces a donor level at 120 meV below the conduction band in the bulk ZnO NR [1,26] and increases the free carrier concentration. The current transport is significantly affected by present traps due to the inherently weak screening of injected and trapped charges in nanorods [27]. Previous studies reported the effect of Al doping on defects present in the ZnO nanostructures [6,28,29,30].

In this paper, we used the chemical bath reaction for the growth of ZnO NR, which is one of the simplest, nontoxic, low-temperature, and low-cost methods. Among the various methods, the spin-coating method was applied to prepare the ZnO seed layer on substrates because of its low cost and easy approach. The electrical properties of undoped and Al-doped ZnO NR were studied by KPFM and temperature-dependent current–voltage (I–V–T) measurements. The analysis of transport processes provided information about the electron traps present in ZnO NRs and the effects related to Al doping.

## 2. Materials and Methods

The following materials and chemicals were used: n-doped Si wafers (~450 µm, 2–7 Ω·cm), deionized water (Milipore, Burlington, NJ, USA), acetone (Merck, Darmstadt, Germany), ethanol (EtOH, aps., Honeywell, Offenbach am Main, Germany), methanol (Honeywell, Offenbach am Main, Germany), hydrofluoric acid 40% (HF, Sigma-Aldrich, Steinheim am Albuch, Germany), zinc acetate dehydrate (Honeywell, Offenbach am Main, Germany), zinc nitrate hexahydrate (Acros Organics, Geel, Belgium), hexamethylentetramine (Sigma-Aldrich, Steinheim am Albuch, Germany), monoethanolamine (MEA, Merck, Darmstadt, Germany), and aluminum nitrate nonahydrate (Honeywell, Offenbach am Main, Germany). All of the materials were analytical grade and used as received without further purification.

ZnO NRs were prepared in two steps. The first step was the deposition of the ZnO seed layer by spin-coating a solution of 0.25 M zinc acetate and MEA in EtOH on previously cleaned (acetone, methanol, and water in ultrasound for 10 min each, followed by a 60 s dip in 2% HF solution and UV ozone cleaner for 10 min as the last step) Si substrates. Si substrates coated with the ZnO seed layer were immersed in an upside-down position in a glass beaker, filled with an aqueous solution of 0.025 M zinc nitrate and 0.025 M hexamethylenetetramine (HMT); for 3 at.% Al doping, aluminum nitrate was added and kept at 85 °C for 1 h. At the end of the growth period, the substrates were taken from the solution and immediately rinsed with deionized water to remove the residuals from the surface and dried in a nitrogen stream.

**Figure 1:**
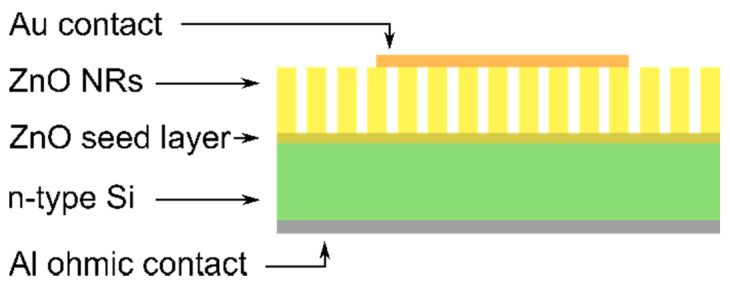
A schematic illustration of prepared Au/ZnO NR/n-Si junction for electrical characterization.

For electrical characterization, metallic contacts were prepared. Aluminum (Al) contacts were deposited on the back surface of the Si substrate, while the top gold (Au) contacts were deposited on the front surface of the samples through a mask with circular openings by vacuum thermal evaporation. The fabricated samples are illustrated in Figure 1.

Temperature-dependent current–voltage (I–V–T) measurements were performed using a 4200-SCS Semiconductor Parameter Analyzer (Keithley, Cleveland, OH, USA). The measurements were carried out in a vacuum, in the temperature range from 100 K to 300 K with 25 K temperature steps. Voltage was applied to the top gold contact using a gold tip probe. The bias voltage was swept from −6 V to 4 V and back.

The morphology of the samples was studied by scanning electron microscopy with a field-emission gun (FEG-SEM, Tokyo, Japan) using JEOL JSM-700F.

The surface roughness and the electrical and potential energy were studied by atomic force microscopy (AFM) in dynamic and KPFM in electric mode, using a Nanosurf CoreAFM device (Nanosurf, Liestal, Switzerland) operating in noncontact dynamic acquisition mode with scan time of 0.78 s, 50% setpoint on a 10 × 10 μm^2^ area, tip voltage of −3V, and Si electrically conductive tip coated with chromium and platinum (ElectricMulti75G, BudgetSensors) under ambient conditions. Images were processed with Gwyddion software (2.60) [31].

XRD measurements were performed using XRD6000 Shimadzu device (Shimadzu, Kyoto, Japan) in Bragg Brentano configuration with CuKα radiation using an acceleration voltage of 40 kV and current of 30 mA in 2θ range 2°–80° with a step size of 0.02° 2θ and a counting time of 0.6 s.

## 3. Results and Discussion

### 3.1. Electron Microscopy Analysis

**Figure 2:**
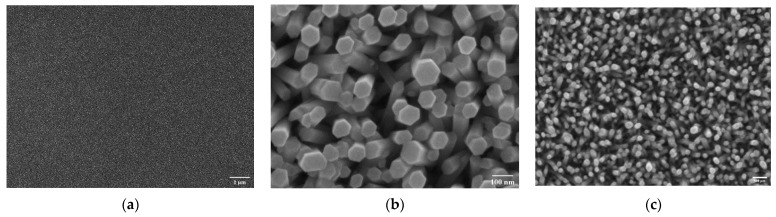
(**a**) Seed layer ZnO; (**b**) as-grown ZnO NR; (**c**) Al-doped ZnO NR.

SEM shows the microscructure of the nanorods. The ZnO NRs grew quite homogeneously with a slight variation in length but within the range of 150–250 nm and a diameter range of 70–100 nm (Figure 2). EDS showed around 1% of Al in the doped ZnO NRs (Table 2). It was assumed that no noticeable change in the morphology could be observed due to the low amount of aluminum doping. However, a notable difference was observed between pure ZnO nanorod samples and those doped with Al, which can be best described as some change in the geometry of the rod tips (Figure 2c). Such a geometry change can occur due to changes during crystallization, i.e., samples show different preferred orientation parameters. The difference seemed to comprise different bending geometry of the NR ends. This behavior is related to the energy levels of certain crystal facets, where the facets with the highest energies want to minimize surface exposure to minimize energy. This behavior is surely the consequence of the Al-doping precursor presence in the growth solution. Statistical analysis of this feature is not feasible using the top-view SEM presented here. Furthermore, statistical analysis of this feature using cross-sections obtained by FIB TEM (focused ion-beam transmission electron microscopy) is simply too time-consuming. Therefore, the differences in morphology were qualified by calculating microstructural parameters from the diffraction analysis.

### 3.2. Atomic Force Microscopy

**Figure 3:**
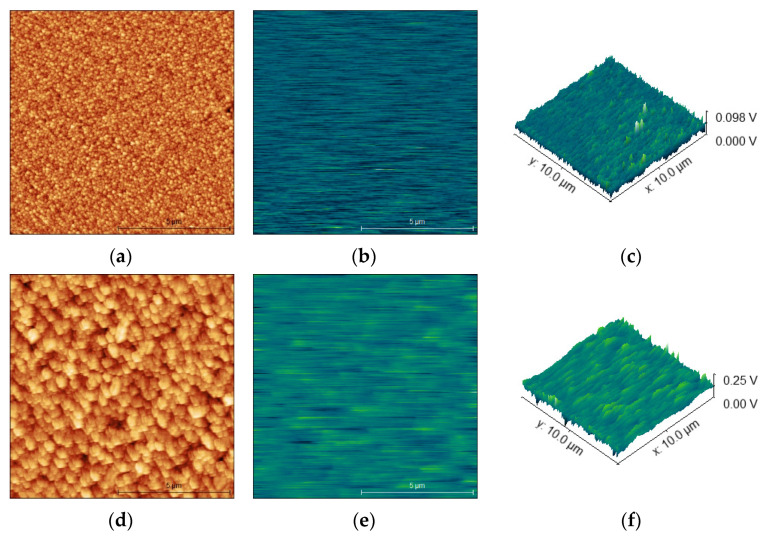
AFM images of ZnO NRs: (**a**) morphology; (**b**,**c**) surface potential scans. AFM images of Al-doped ZnOs NR: (**d**) morphology; (**e**,**f**) surface potential scans.

AFM analysis showed slight variations in the microstructure (Figure 3a,d) of the ZnO NRs and Al-doped ZnO NRs, consistent with the SEM images. In addition, the surface roughness of the samples also showed an increase in value; the undoped sample had a value of Sq 30.81 nm and the doped sample had a value of Sq 49.35 nm, which can be attributed to the addition of Al to the growth solution.

Electrical (KPFM) mode measurements (Figure 3b,c,e,f) also showed a difference in the surface potential of the samples: the undoped sample had values in the range up to 0.098 V, while the Al-doped sample had values in the range up to 0.250 V, further confirming successful Al doping.

### 3.3. Diffraction Analysis

**Figure 4:**
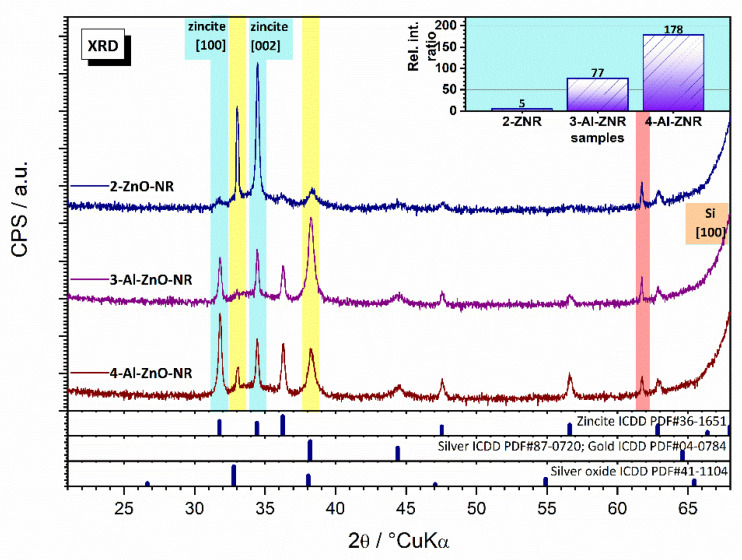
Diffractograms and microstructural parameters of pure ZnO NRs and Al-doped ZnO NRs.

All samples yielded qualitatively the same crystal phase composition with structural differences among the present phases and some semiquantitative differences (Figure 4). The major phase was zincite ICDD PDF#36-1451. The samples seemed to have the same amount of zincite quantitatively. Apparently, the intense (002) zincite reflex does not reflect the higher content of zincite; rather, it suggests the presence of the preferred orientation of zincite. Such a strong intense (002) zincite reflex correlates well with the growth of zincite nanorods. From the change in the mutual relative ratio of zincite (100) to (002) reflex, we can quantify the extent of structural changes (Figure 4 Inset). Specifically, it seems that the aspect ratio of zincite nanorods differed in the samples, affecting the preferred orientation. One-dimensional growth was the strongest for Sample 1 but weakest for Sample 3. The differences between samples seemingly occurred due to the addition of 3% Al. The addition of Al precursor to the ZnO NR growth medium obviously disrupted the 1D growth to some extent. The strongest reflex was the (100) reflex, which was assigned to n-Si, used as the wafer substrate for zincite nanorod growth. From the constituents, the presence of gold ICDD PDF#04-0784 and silver ICDD PDF#87-0720 was observed. Gold was sputtered to ensure electric contact at the surface, while silver paste was used to fix the substrate’s bottom contact. Both phases yielded similar structural features, as visible from the heavily overlapped diffraction pattern. Therefore, it is not possible to deconvolute and distinguish the contribution from Au and Ag. Surprisingly, the presence of a silver oxide ICDD PDF#41-1104 phase was observed. The semiquantitative correlations for Au–Ag–Ag_2_O phases were very interesting. Specifically, only very similar positioning of the specimens in the XRD holder can control the quantity of signal collected from gold sputtered at the surface. Therefore, we can assume that the gold signal does not change significantly. However, the quantity of signals collected from the underlying silver cannot be controlled. As such, the proportional increase in reflexes (111) and (200) occurred because of different amounts of signal collected from silver. In addition, Ag (200) and Au (200) are overlapped by Ag_2_O (200). However, the extent of contribution could hardly be observed, even for Sample 2 where Ag_2_O presence could not be neglected. Another explanation for such behavior may be found in the preferred orientation, which is common in such films. This is especially visible for zincite, as previously mentioned. The consequence of this growth mechanism change is in concordance with SEM results, as already explained above.

### 3.4. Optical and Electrical Analysis

**Figure 5:**
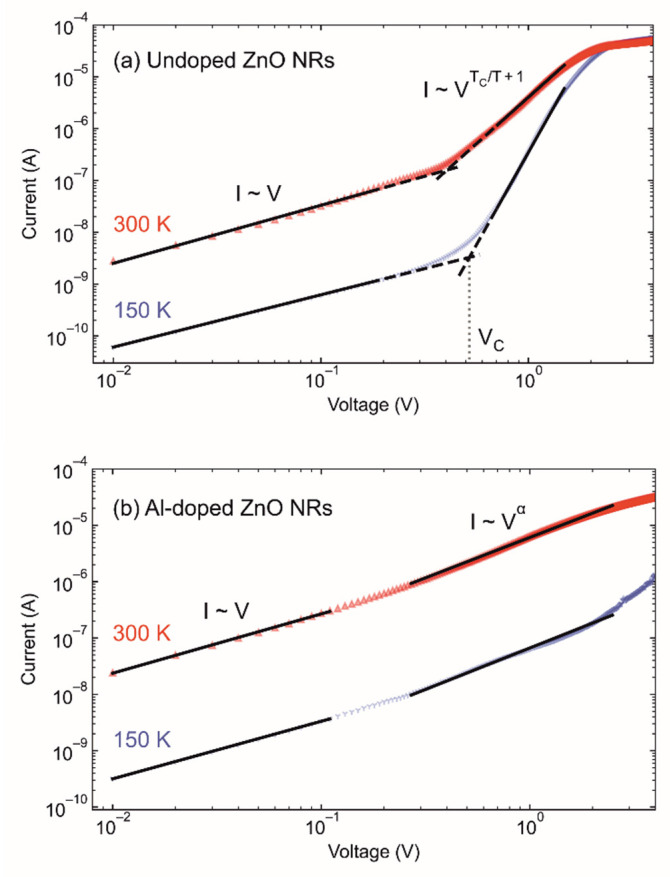
The forward I–V–T characteristics of the samples with (**a**) undoped and (**b**) Al-doped ZnO NRs measured at different temperatures. Two linear regions are observed in both cases.

The optical properties are an important part of ZnO thin-film investigations. The literature reports bandgaps for pure ZnO NR at about 3.37 eV, and doping modification by Al moves the bandgap value toward 2.98 eV [32,33]. Following the same type of deposition, we expected similar values. However, the confirmation of bandgap values for our samples was omitted due to the use of Si wafers as substrates to focus on electric performance. The forward I–V characteristics at selected temperatures of the samples with undoped and Al-doped ZnO NRs are shown on the log–log plot in Figure 5. We can clearly distinguish two linear regions in both cases. For the lower voltages, I–V characteristics were well described by the ohmic transport process (undoped and Al-doped ZnO NRs), whereas, for the higher voltages, a significant difference between undoped and Al-doped ZnO NRs was detected.

At the higher voltages (V > V_c_) the I–V characteristics for undoped ZnO NRs (Figure 5a) were described by the space charge-limited current (SCLC) transport processes. In the case of Al-doped ZnO NR (Figure 5b), the I–V characteristics for voltages > 0.3 V were described by the relation I~V^α^.

Temperature-induced changes in I–V characteristics were more pronounced in the case of undoped ZnO NRs. The crossover point (critical voltage, V_c_) was clearly defined and shifted to higher voltages with decreasing temperature. A sharp transition between the two linear regions was not detected for Al-doped ZnO NRs.

**Figure 6:**
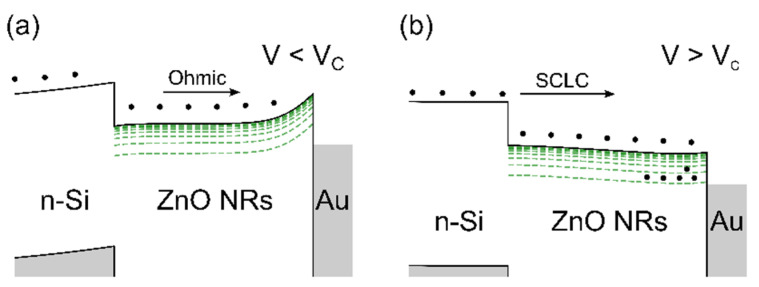
Schematic illustration of Au/ZnO NR/n-Si junction energy band diagram calculated using the SCAPS [34] simulation program. The electron affinities of Si (4.05 eV) and ZnO (4.35 eV), bandgaps of Si (1.12 eV) and ZnO (3.25 eV), and work function of Au (5.1 eV) were used in the calculations [35]. The trap states below the conduction band are illustrated in green. (**a**) Ohmic current due to thermally generated free carriers in the ZnO NRs. (**b**) SCLC determined by trapped charge near the injecting ZnO NR/n-Si contact.

In order to understand the influence of Al doping on the transport properties of ZnO NRs, we analyzed the I–V characteristics of undoped and Al-doped ZnO NRs using the ohmic and SCLC transport processes. The basic energy band diagrams for these two processes are given in Figure 6.

At lower voltages, the current scales linearly with voltage (as seen in Figure 5a,b).

(5) $$I=\sigma \frac{S}{L}V$$

where S is the area of the Au electrical contact, L is the width of the ZnO NR layer, and σ is the conductivity of the ZnO NR layer. The increase in ohmic current with the temperature is well described by an increase in thermally generated free carrier concentration. Al doping increased the conductivity of the ZnO NR layer by an order of magnitude. It is known that undoped ZnO NRs are n-type due to intrinsic defects such as oxygen vacancies and zinc interstitials [26]. The estimated values for conductivity for undoped and Al-doped ZnO NRs measured at 150 K and 300 K are listed in Table 3. As previously reported, the incorporation of Al^3+^ ions at the Zn lattice site during the growth of ZnO NRs leads to an increase in free carrier concentration and, consequently, to an increase in conductivity of ZnO NR [18,26].

**Figure 7:**
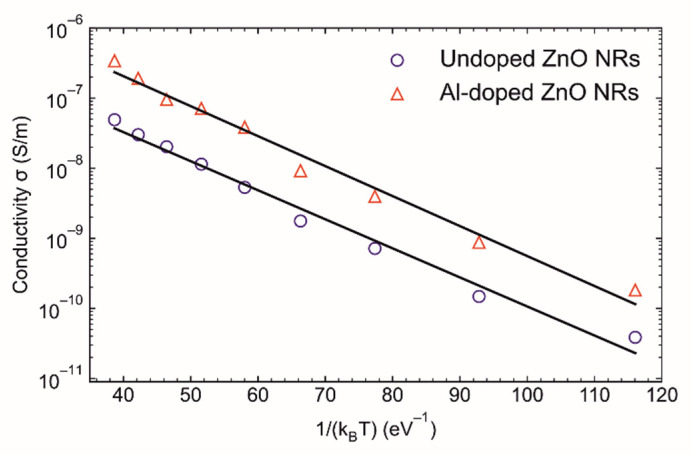
Arrhenius plot of electrical conductivity for undoped and Al-doped ZnO NRs.

Figure 7 shows the Arrhenius plot of electrical conductivity for undoped and Al-doped ZnO NRs. The apparent activation energies derived from this plot were 0.095 eV and 0.099 eV for the undoped and Al-doped ZnO NRs, respectively. The estimated activation energies are close to previously reported values for ZnO NRs [36]; the differences are due to the different growth methods and conditions. The observed linearity indicates that a thermally activated transport process dominated in the measured temperature range (100–300 K).

At higher voltages, the transition to the SCLC regime occurred due to the injected carrier concentration becoming comparable to the thermally generated free carrier concentration in undoped ZnO NR.

Electrons are easily injected into the ZnO NR layer owing to significant conduction band discontinuity ΔE_C_ = 0.3 eV [37] at the ZnO NR/n-Si contact. The traps in the ZnO NR layer capture electrons; hence, the negative space charge density increases with an increase in positive bias. The negative trapped charge and the positive charge on the Au contact introduce an electric field that affects the current flowing in response to the applied voltage (as schematically represented in Figure 6). Therefore, the SCLC analysis provides useful information regarding the concentration of traps in the ZnO NR layer and their energy distribution [38,39,40].

In the case of the undoped ZnO NRs (Figure 5a), the I–V–T characteristic at voltages above 0.5 V (V_C_) is well described by the following relation [38]:

(6) $$I={e\mu N}_{C}{\left(\frac{\varepsilon }{{\mathrm{eN}}_{T}}\right)}^{T_{C}/T}\frac{V^{T_{C}/T+1}}{L^{2T_{C}/T+1}}$$

Deduced in the case of the exponential density of trap energy levels below the bottom of the conduction band,

(7) $$n_{T}\left(E\right)=\frac{N_{T}}{E_{T}}\exp\left(-\frac{E_{C}-E}{E_{T}}\right)$$

where $E_{T}=k_{B}T_{C}$ is the characteristic trap energy, ε is the dielectric constant, e is the elementary charge, μ is the electron mobility, $N_{C}$ is the effective density of states in the conduction band, and $N_{T}$ is the total trap concentration. The characteristic trap energy E_T_ = 79 meV is determined from the temperature dependence of voltage exponent α = T/T_C_ + 1, while the total trap concentration N_T_~5 × 10^15^–10^16^ cm^−3^ is estimated from the temperature dependence of the prefactor in Equation (6). The estimate of total trap concentration assumed a ZnO NR length of 150–250 nm (estimated from SEM and AFM), a relative dielectric constant ε_r_ = 3 of ZnO NRs [41], and a temperature-independent μN_C_. It is believed that the characterized traps correspond to surface states [42] or electron traps in the bulk ZnO NRs [43].

As already mentioned, the crossover to the SCLS regime was not observed for Al-doped ZnO NRs. In the case of Al-doped ZnO NRs (Figure 5b), the increase in the forward current was more gradual, and the current did not reach the saturation current value unlike the case of the undoped sample.

In fact, the current followed the relation I~V^α^ with exponent α = 1.5 showing only a small deviation from the ohmic behavior. Such a dependence was also preserved at the lower temperatures.

It should be noted that the increase in the trap concentrations or the introduction of donors could shift the trap-filled limit to higher voltages, and a crossover (as observed for undoped ZnO NR) could occur at voltages greater than the measured voltage range (>4 V). We believe that Al doping introduced traps i.e., energy levels, and those traps have a strong influence on SCLS transport properties. Further studies are needed for a better understanding of Al doping influence on the transport properties of ZnO NR, especially due to the ambiguous contribution of changes in ZnO NR geometry [44].

## 4. Conclusions

In this work, we investigated the most important electric properties of undoped and Al-doped (3 at.%) ZnO nanorods prepared via a facile two-step wet-chemistry technique. Firstly, ZnO thin films were deposited on silicon wafers by spin-coating; secondly, ZnO nanorods (growth solution contained hexamethylenetetramine and zinc nitrate hexahydrate) and Al-doped ZnO NRs (growth solution additionally contained 3 at.% aluminum nitrate nonahydrate) were grown via a chemical bath method.

The SEM micrographs confirmed that closely packed arrays of vertically aligned nanorods with approximately the same lengths were achieved for all synthesis conditions. SEM revealed that the geometry of the Al-doped rods marginally changed. XRD results revealed that the as-synthesized nanorods were highly crystalline with preferential growth along the c-axis, as expected for ZnO thin films. Phase separation and impurities were not observed in the XRD results. Samples with Al doping showed preferred orientation to a different extent, suggesting a different effect on the ZnO lattice. Calculated microstructural parameters confirmed the SEM and AFM observations; AFM analysis confirmed the slight differences in morphology that were observed in SEM. The KPFM electrical mode of AFM showed larger differences between sample groups, highlighting the successful synthesis and Al doping, which was subsequently confirmed by the I/V measurements.

The electrical properties of undoped and Al-doped ZnO nanorods were thoroughly studied. The understanding of the control achieved by means of intentional doping is crucial for the further development and application of electronic devices based on ZnO nanorods. For the lower voltages, the I–V characteristics of undoped and Al-doped ZnO nanorods both followed the ohmic regime, whereas Al-doping influenced the transport properties at higher voltages. Moreover, Al doping increased the conductivity of ZnO nanorods by an order of magnitude. Nevertheless, the contribution of ZnO nanorod geometry to overall electric properties still requires further investigation. Specifically, the growth of the rods is a chemical process where the system wants to reach an energetically more favorable state by favoring directional growth, i.e., nanorods. Researchers have tried to describe and understand the system to consequently have control over the growth parameters. Using various approaches, it is possible to control the shape, length, alignment, density, diameter, distances, and branching parameters to some extent. However, until now, under pragmatic conditions, it was not possible to mitigate the relative difference in lengths of the nanorods, as well as slight tilting from the perpendicular orientation to the substrate. This is particularly true for the case when modifications are implemented, as in our case. Thus, the intended application requires interfacing to other layers where the mentioned geometric discrepancies still need to be addressed. Thus, this area is still interesting for researchers.

## Figures and Tables

**Table 1:** Growth of ZnO NRs using different techniques.

Technique	Specifications	Reference
Chemical vapor deposition	Accurate growth control, high T, carrier gas	[9]
Thermal evaporation	Simple, no catalysts, high temperature	[10]
RF magnetron sputtering	High sample purity, low cost, low pressure	[11]
Pulsed laser deposition	Gas pressure control, high T, low pressure	[12]
Spray pyrolysis	Does not require high-quality targets	[13]

**Table 2:** EDS analysis.

Element	Mass (%)	MassNorm. (%)	Atom (%)
Oxygen	4.85	4.72	8.11
Aluminum	0.91	0.88	0.90
Silicon	94.28	91.65	89.83
Zinc	2.83	2.75	1.16
Total	102.87	100.00	100.00

**Table 3:** The electrical conductivity σ of undoped and Al-doped ZnO NRs.

Sample	*σ* (mS·cm^−1^) at 300 K	*σ* (mS·cm^−1^) at 150 K
Undoped ZnO NR	3–5 × 10^−5^	5–9 × 10^−7^
Al-doped ZnO NR	2–4 × 10^−4^	3–5 × 10^−6^
